# Augmented Cardiac Hypertrophy in Response to Pressure Overload in Mice Lacking ELTD1

**DOI:** 10.1371/journal.pone.0035779

**Published:** 2012-05-11

**Authors:** Jinfeng Xiao, Hong Jiang, Rui Zhang, Guangpu Fan, Yan Zhang, Dingsheng Jiang, Hongliang Li

**Affiliations:** 1 Department of Cardiology, Renmin Hospital of Wuhan University, Wuhan, People’s Republic of China; 2 Cardiovascular Research Institute, Wuhan University, Wuhan, People’s Republic of China; 3 Department of Thoracic and Cardiac Surgery, Tongji Hospital, Tongji Medical College, Huazhong University of Science and Technology, Wuhan, People’s Republic of China; Cardiovascular Research Institute Maastricht, Maastricht University, The Netherlands

## Abstract

**Background:**

Epidermal growth factor (EGF), latrophilin and seven transmembrane domain-containing protein 1 (ELTD1) is developmentally upregulated in the heart. Little is known about the relationship between ELTD1 and cardiac diseases. Therefore, we aimed to clarify the role of ELTD1 in pressure overload–induced cardiac hypertrophy.

**Methods and Results:**

C57BL/6J wild-type (WT) mice and ELTD1-knockout (KO) mice were subjected to left ventricular pressure overload by descending aortic banding (AB). KO mice exhibited more unfavorable cardiac remodeling than WT mice 28 days post AB; this remodeling was characterized by aggravated cardiomyocyte hypertrophy, thickening of the ventricular walls, dilated chambers, increased fibrosis, and blunted systolic and diastolic cardiac function. Analysis of signaling pathways revealed enhanced extracellular signal-regulated kinase (ERK) and the c-Jun amino-terminal kinase (JNK) phosphorylation in response to ELTD1 deletion.

**Conclusions:**

ELTD1 deficiency exacerbates cardiac hypertrophy and cardiac function induced by AB-induced pressure overload by promoting both cardiomyocyte hypertrophy and cardiac fibrosis. These effects are suggested to originate from the activation of the ERK and JNK pathways, suggesting that ELTD1 is a potential target for therapies that prevent the development of cardiac disease.

## Introduction

Cardiac hypertrophy, an increase in heart mass, reflects a remodeling process in various cardiac diseases [1]. The initial stage of cardiac hypertrophy is an adaptive process; however, sustained hemodynamic overload leads to uncompensated hypertrophy associated with increased interstitial fibrosis and impaired cardiac dysfunction; and this uncompensated hypertrophy represents one of the major risk factors for heart failure. Initiating stimuli are sensed through an array of membrane-bound G-protein-coupled receptors (GPCRs) and regulate remodeling by various signaling pathways. Mitogen-activated protein kinases (MAPKs), along with phosphoinositide 3-kinase (PI3K) – Akt are well-studied family of proteins that play an integral role in these signaling events [2], [3]. However, the mechanisms through which hypertrophy eventually leads to heart failure remain poorly understood.

ELTD1 is composed of an extracellular domain with epidermal growth factor (EGF)-like repeats and a seven-transmembrane domain (TMD7) followed by a short cytoplasmic tail [4]. The mRNA level of ELTD1 is up regulated postnatally both in rats and human hearts [4]. The growth of heart is contributed mainly to proliferation of cardiomyocytes in the initial stage and hypertrophy of cardiomyocytes accompanied by remodeling of the nonmyocyte in mature myocardium. The up-regulation of ELTD1 expression in rat and human hearts couples with the switch of myocardium from hyperplastic to hypertrophic growth [4] ,suggesting ELTD1 to be an important effector in this process. Furthermore, the extracellular domain of rat ELTD1 possesses several common protein kinase phosphorylation sites, and the short cytoplasmic tail carries a tyrosine kinase phosphorylation site that could couple to a tyrosine kinase signaling pathway [4]. The putative phosphorylation site could play a part in signal transductions employing tyrosine kinases such as MAPKs pathway [5] and serve as a scaffold for the phosphotyrosine-dependent complex [6]. The overall structure of ETLD1 suggests that the protein might participate in both cell surface events such as cell-cell interaction and in signaling cascades in cardiac development. However, no research has been conducted on the role of ELTD1 in cardiac diseases using animal models to date [4]. Therefore, the aims of this study were to determine if deletion of the ELTD1 gene affects pressure overload-induced cardiac hypertrophy and to identify the processes that underlie ELTD1-related differences in the hypertrophic growth response.

## Materials and Methods

### Animals and Aortic Banding

All animal experimental protocols were approved by the Animal Care and Use Committee of Renmin Hospital of Wuhan University(protocol number: 00022980), and the protocols were performed in accordance with the Guide for the *Care and Use of Laboratory Animals* published by the US National Institutes of Health (NIH Publication No. 85–23, revised 1996). C57BL/6 (WT) mice and ELTD1 knockout (KO) mice (C57BL/6J background) were provided by the European Mouse Mutant Archive. Their genotypes were confirmed by PCR (data not shown). The KO mice were viable and showed normal atrial and ventricular morphology and a normal heart rate, systolic pressure, contractility (+dP/dt), and relaxation (−dP/dt) at baseline (data not shown). KO and WT male mice, aged between 8 and 10 weeks, were randomly assigned to thoracic aortic banding (AB) group or sham group respectively, and the numbers of mice are 15,15,20,25 in WT-Sham, KO-Sham, WT-AB, and KO-AB groups respectively. AB surgeries are conducted as previously described [7]. Briefly, mice were anesthetized with pentobarbital sodium (90 mg/kg, i.p.) and ventilated with room air using a small animal ventilator (model VFA-23-BV, Kent Scientific, USA). Mice were kept warm on a heating pad until they regained consciousness. After conducting a thoracotomy in the second or third intercostal, the descending aortic was ligated by tying a 7–0 silk suture against a 26-gauge needle. The same procedure was used for the sham operation without ligation of the aorta.

### Human Ventricular Samples

We analyzed the protein level of ELTD1 of myocardial samples taken from failing human hearts and healthy controls. Of these, samples from 4 failing hearts were prepared during orthotropic heart transplantation representing myocardium from end-stage heart failure patients (NYHA IV).The failing hearts were developed from dilated-hypokinetic evolution of hypertrophic cardiomyopathy, and is characterized by cavity dilation and increase of heart mass (data not shown). All patients had no signs of familial etiology. Control samples were obtained from the left ventricles of the 4 normal heart donors. After extraction of the heart, samples were immediately frozen in liquid nitrogen and stored at −80°C until their processing. The protocols were reviewed by the local Ethical Committee (Renmin Hospital of Wuhan University Human Research Ethics Committee, Wuhan, China), and the experiments were conducted in accordance with the Tri-Council Policy Statement: Ethical Conduct for Research Involving Humans. Informed written consent was obtained from all subjects.

### Histological Analysis

The hearts were harvested and randomly assigned for bio-molecular and histological analyses. For histological analysis, the hearts were fixed in 10% formalin, embedded in paraffin, and sectioned at 1-mm intervals for staining. A single myocytes was measured using images captured from hematoxylin and eosin (H&E)–stained sections. For digital measurements of cardiomyocyte cross-sectional areas (CSAs), 150 cells per genotype were traced using Image Pro Plus (version 6.0, Media Cybernetics, USA). The cardiac collagen volume was calculated as the ratio of the sum of the total collagen area to the sum of the total collagen and no collagen areas in the entire visual field of the section as determined by picrosirius red (PSR) staining.

### Echocardiography

Transthoracic echocardiography was performed using MylabTM30CV (ESAOTE S.p.A.) with a 10-MHz linear array ultrasound transducer before and 28 days after surgery. Measurements were made by an observer blinded to the experimental group. The chamber diameters of left ventricular (LV) were measured at end-systole (LVESD) and end-diastole (LVEDD). Fractional shortening (FS) and the ejection fraction (EF) were calculated as previously described [7].

### Hemodynamic Measurements

Hemodynamic measurements were obtained 28 days after AB using a 1.4-French high-fidelity catheter (SPR-839, Millar Instruments, Houston, TX, USA), as described previously [7]. In short, the catheter was inserted into the right carotid artery and advanced into the left ventricle after the animals were anesthetized to acquire signals. After having been stabilized for 15 minutes, the signals were continuously recorded using a Millar Pressure-Volume System (MPVS-400, Millar Instruments, Houston, TX, USA). PVAN software (Millar Instruments, USA) was used for the subsequent analysis of the pressure-volume loops (PV-loop).

### Quantitative Real-time RT-PCR

Real-time PCR was performed to quantify relative transcript levels in various groups. cDNA was reverse transcribed using oligo (dT) primers with the Advantage RT-for-PCR Kit (BD Biosciences). A real-time Roche LightCycler PCR system was used to run PCR reactions with the SYBR Green PCR Master Mix (Applied Biosystems) The real-time PCR results from each primer pair were normalized to those of glyceraldehyde-3-phosphate dehydrogenase (GAPDH) gene expression and were compared across conditions. The primer sequences used for RT-PCR are shown in Table 1.

**Table 1:** A list of the primers used in this study.

Gene Name	Forward (5′–3′)	Reverse(5′–3′)
ELTD1	GGACCAGTTACCGACAAATCACA	GATGAGGATAGCAAGGGACCAAT
α-MHC	GTCCAAGTTCCGCAAGGT	AGGGTCTGCTGGAGAGGTTA
β-MHC	CTTCACGGGCACCCTTGGA	ACCTGCTAGACCACCTGGAG
*Anp*	ACCTGCACCACCTGGAGGAG	CCTTGGCTGTTATCTTCGGTACCG
*Bnp*	GAGGTCACTCCTATCCTCTGG	GCCATTTCCTCCGACTTTTCTC
*Serca2α*	CATTTGCATTGCAGTCTGGAT	CTTTGCCATCCTACGAGTTCC
*Acta1*	GTGAGATTGTGCGCGACATC	GGCAACGGAAACGCTCATT
*Tgf-β1*	ATCCTGTCCAAACTAAGGCTCG	ACCTCTTTAGCATAGTAGTCCGC
*Tgf-β2*	TCGACATGGATCAGTTTATGCG	CCCTGGTACTGTTGTAGATGGA
*Col1α1*	CCTCAA GGG CTC CAA CGA G	TCA ATC ACT GTC TTG CCC CA
*Col3α1*	ACGTAGATGAATTGGGATGCAG	GGGTTGGGGCAGTCTAGTC
*Fibronectin*	CCGGTGGCTGTCAGTCAGA	CCGTTCCCACTGCTGATTTATC
*Ctgf*	TGACCCCTGCGACCCACA	TACACCGACCCACCGAAGACACAG
*Mmp-2*	CTTTGCCATCCTACGAGTTCC	CCATCAAACGGGTATCCATCTC
*Mmp-9*	CGGACCCGAAGCGGACAT	GGGGCACCATTTGAGTTT

### Protein Analyses

Total protein samples were extracted from left ventricles, subjected to SDS–PAGE, and then eletrotransferred to nitrocellulose membranes (#IPFL00010, Millipore). The Western blots were scanned with a two-color infrared imaging system (Odyssey, LI-COR). The GAPDH protein was used as the endogenous control. Antibodies against the following proteins were purchased from Cell Signaling Technology: p-MEK1/2 (#9154), T-MEK1/2 (#9122), p-ERK1/2 (#4370), T-ERK1/2 (#4695), p-p38 (#4511), T-p38 (#9212), p-JNK1/2 (#4668), T-JNK 1/2(#9258), p-Akt (#4060), T-Akt (#4691), p-GSK3β (#9322), T-GSK3β (#9315), p-mTOR (#2974), T-mTOR (#2972), and GAPDH (#2118). The antibody for ELTD1 (sc-46947) was purchased from Santa Cruz Biotechnology.

### Statistical Methods

Results are presented as the mean ± SEM. Statistical comparisons were performed using SPSS (Statistical Package for the Social Sciences) 17.0 software with ANOVA either alone or followed by Tukey’s multiple-comparison test. Student’s t-tests were used to compare means between two groups. P<0.05 was considered statistically significant.

## Results

### ELTD1 Protein Levels Decrease in the Ventricles of Patients with End Stage Heart Failure

**Figure 1:**
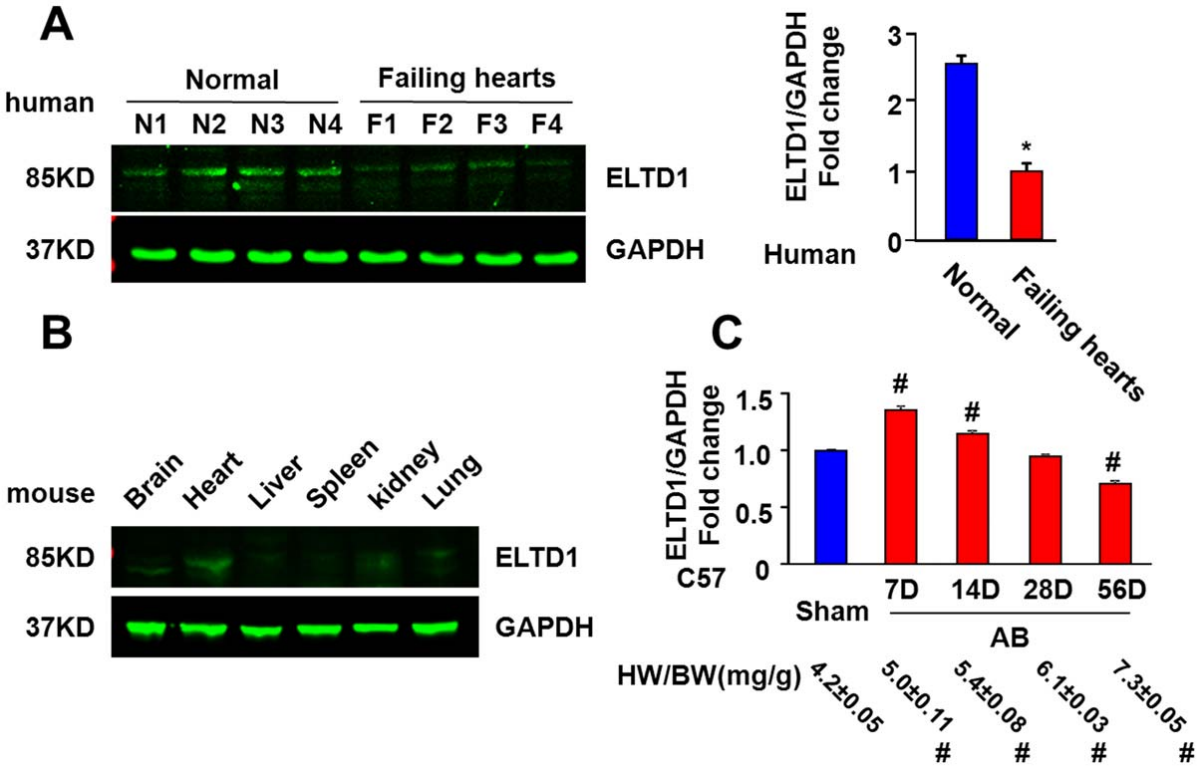
ELTD1 expression in normal and hypertrophied ventricles. A, Representative and statistical data of western blotting for protein levels of ELTD1 with glyceraldehyde phosphate dehydrogenase (GAPDH) as loading control (n = 4). *P<0.05 vs. normal donators. Left, representative blots. Right, quantitative results. B, Western blotting for levels of ELTD1 in mice at different tissue indicated (n = 4). C, Real-time PCR for ELTD1 and heart weight/body weight (HW/BW) in WT control mice after AB at time points indicated (n = 6). Data represent as mean±SEM. Up, ratios indicate mRNA levels normalized to GAPDH. #P<0.01 vs. the sham values.

We examined ELTD1 expression in human ventricular samples from patients undergoing a heart transplant and from healthy donors. Ventricular tissue from failing hearts exhibited a 2- to 3-fold decrease in the ELTD1 protein level relative to those from normal donors (Figure 1A).

### ELTD1 Gene Expression in Mice Ventricles before and after AB

By assessing the expression pattern in various tissues, we found a high protein level of ELTD1 in the heart of C57BL/6 mice (Figure 1B). Next, we examined the relative levels of ELTD1 by real-time PCR using samples from ventricles collected 7, 14, 28, and 56 days post AB or immediately after sham surgery. There is a trend of increase in heart mass during that time (Figure 1C), and the wall thickness and chamber diameter increase correspondingly (data not shown). The levels of ELTD1 mRNA reached a maximum at 7 days after AB and then decreased (Figure 1C).

### Effect of ELTD1 Deficiency on Cardiac Hypertrophy

**Figure 2:**
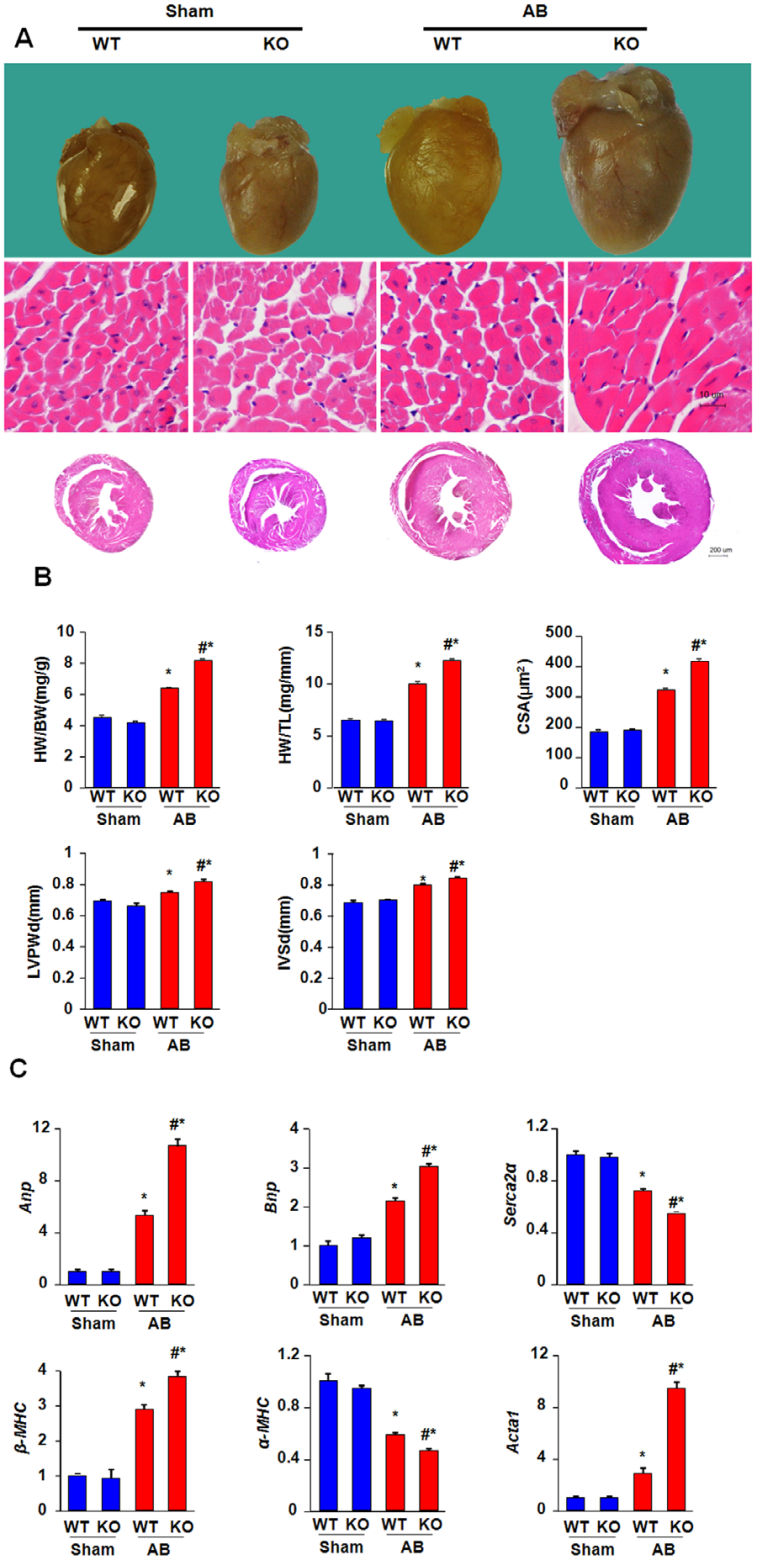
The effects of ELTD1 on cardiac hypertrophy 28 days post-surgery. A, Representative gross heart, wall thickness and HE staining in WT and KO mice B, Statistical results of the HW/BW, HW/TL and myocytes cross-sectional areas(CSA) of indicated groups(n = 8 mice per group, and the number of myocytes is 150 per group). C, Parameters of wall thickness by statistical analysis of LVPWD and IVSD (n = 8). D, Analysis of *Anp, Bnp, α-MHC, β-MHC, Serca2α,* and *Acta1* in WT and KO mice by real-time PCR analysis (n = 6). For B, C and D, Data represent as mean±SEM. *P<0.01 vs. corresponding sham; #P<0.01 vs. WT/AB after AB.

We measured the heart weight (HW), lung weight (LW), body weight (BW), and tibia length (TL) at 28 days after surgery. In the sham operation group, the ratios of the heart weight to body weight (HW/BW) or to tibia length (HW/TL) were not different between the KO and WT mice. AB induced a significant increase in HW/BW and HW/TL in both WT and KO mice, and the increase was more pronounced in KO mice than in WT mice (Figure 2B). Morphometric assessment of H&E-stained LV sections revealed that the CSA tended to be larger in KO mice than in WT mice after AB (Figure 2B). Echocardiography confirmed left ventricular hypertrophy in the KO mice, as measured by the left ventricle posterior wall thickness (LVPWT) and interventricular septal thickness at end-diastole (IVSD). The wall thickness was similar in sham-operated KO and WT mice. AB significantly increased the LVPWT and IVSD in both WT and KO mice. ELTD1 deficiency augmented the increases in the LVPWD and IVSD after AB (Figure 2C). Expression of the hypertrophy genes, atrial natriuretic peptide (*Anp*), brain natriuretic peptide (*Bnp*), α-myosin heavy chain (*α-MHC*), *β-MHC*, actin α1 skeletal muscle (*Acta1)* and sarco/endoplasmic reticulum Ca2+-transport ATPase2α (*Serca2α*) correlated with the above-described results. *Anp, Bnp, α-MHC*, and *Acta1* were strongly upregulated after 28 days of AB in WT mice. This up-regulation was paralleled by a strong reduction in *β-MHC* and *Serca2α* expression, and these changes were even evident in KO mice in response to AB (Figure 2C).

### LV Function and Chamber Diameter

To estimate the cardiac function and chamber diameter, echocardiography and PV loop measurements were performed before mice were euthanized 28 days after the operations. As shown in Table 2, the chamber diameter and LV function parameters did not differ between sham-operated WT and KO mice. The LV chamber was enlarged after AB as indexed by LVEDD, LVESD, and the end-diastolic and end-systolic volumes (ESV and EDV, respectively) and was increased even more in KO mice than in WT mice. Accordingly, the end-systolic and end-diastolic pressures (ESP and EDP, respectively) were higher in KO mice than in WT mice after AB. Indeed, AB significantly impaired cardiac function as evidenced by the markedly reduced dP/dtmax and dP/dtmin as compared to sham-operation (Table 2). ELTD1 deficiency aggravated the depression in the systolic function (dP/dtmax, EF, FS, cardiac output and stroke volume) after pressure overload, and similar results were observed for the diastolic function (Tau_w and dP/dtmin) and ventricular afterload (indexed by Ea, arterial elastance) (Table 2). These effects were associated with an increase in the wet lung weight (LW), and increases in the wet LW divided by the body weight or the tibia length indicated more pulmonary congestion in the KO mice.

**Table 2:** Hemodynamic parameters in mice 28 days post-surgery.

	Sham		AB	
Parameter	WT(n = 8)	KO(n = 8)	WT(n = 8)	KO(n = 8)
BW(g)	26±0.64	28.01±0.43	27.35±0.53	26.91±0.39
LW(mg)	135.4±3.31	131.4±4.06	158.5±5.72*†	264.4±27.74#*†
LW/BW(mg/g)	5.22±0.14	4.69±0.11	5.79±0.16*†	9.91±1.14#*†
LW/TL(mg/mm)	7.49±0.12	7.22±0.20	9.03±0.34*†	14.75±1.57#*†
***ECHO***				
LVEDd(mm)	3.63±0.06	3.56±0.06	4.31±0.04*†	4.78±0.06#*†
LVESd(mm)	2.07±0.05	2.12±0.05	2.92±0.05*†	3.64±0.08#*†
FS (%)	43.13±0.92	40.63±0.67	32.36±0.78*†	23.92±0.72#*†
***Hemodynamics***				
HR (min–1)	472.6±10.1	464.5±13.3	455.8±13.5	481.0±15.2
ESP (mmHg)	101.4±3.7	104.2±4.0	149.4±4.3*†	176.5±3.2#*†
EDP (mmHg)	12.0±0.8	11.7±1.1	16.3±1.0	28.9±3.3#*†
ESV (µl)	15.0±0.8	13.5±1.0	19.9±0.6*†	24.3±1.0#*†
EDV (µl)	29.2±1.1	29.1±0.5	32.6±0.3*†	36.4±0.7#*†
***Systolic function***				
dP/dtmax (mmHg/s)	9856.6±471.4	9439.7±398.4	8130.6±400.3	7327.4±463.8#*†
Ea (mmHg/µl)	5.4±0.3	5.8±0.5	12.3±0.6*†	26.9±5.4#*†
EF (%)	54.9±1.4	52.5±3.5	39.3±2.1*†	21.2±2.3#*†
CO (µl/min)	9022.3±366.6	8426.7±656.0	6177.6±223.6*†	3465.8±481.6#*†
Stroke volume (µl)	19.0±0.5	18.7±1.7	13.7±0.8*†	7.4±1.1#*†
***Diastolic function***				
dP/dtmin (mmHg/s)	−9075.4±355.3	−9431.5±289.4	−8478.6±161.3*†	−5530.6±347.7#*†
Tau_w(ms)	6.0±0.3	6.1±0.4	9.2±1.5*†	13.7±1.0#*†

HR, heart rate; ESP, end-systolic pressure; EDP, end-diastolic pressure; ESV, end-systolic volume; EDV, end-diastolic volume; Ea, arterial elastance; EF, ejection fraction; CO, cardiac output; dp/dtmax, maximal rate of pressure development; dp/dtmin, maximal rate of pressure decay; Tau_w, time constant of isovolumic pressure decay.

* P<0.05 vs. WT/sham; †P<0.05vs.KO/sham; #P<0.05 vs. WT/AB after AB.

### ELTD1 Deficiency Exacerbates the Fibrotic Response Induced by Pressure Overload

**Figure 3:**
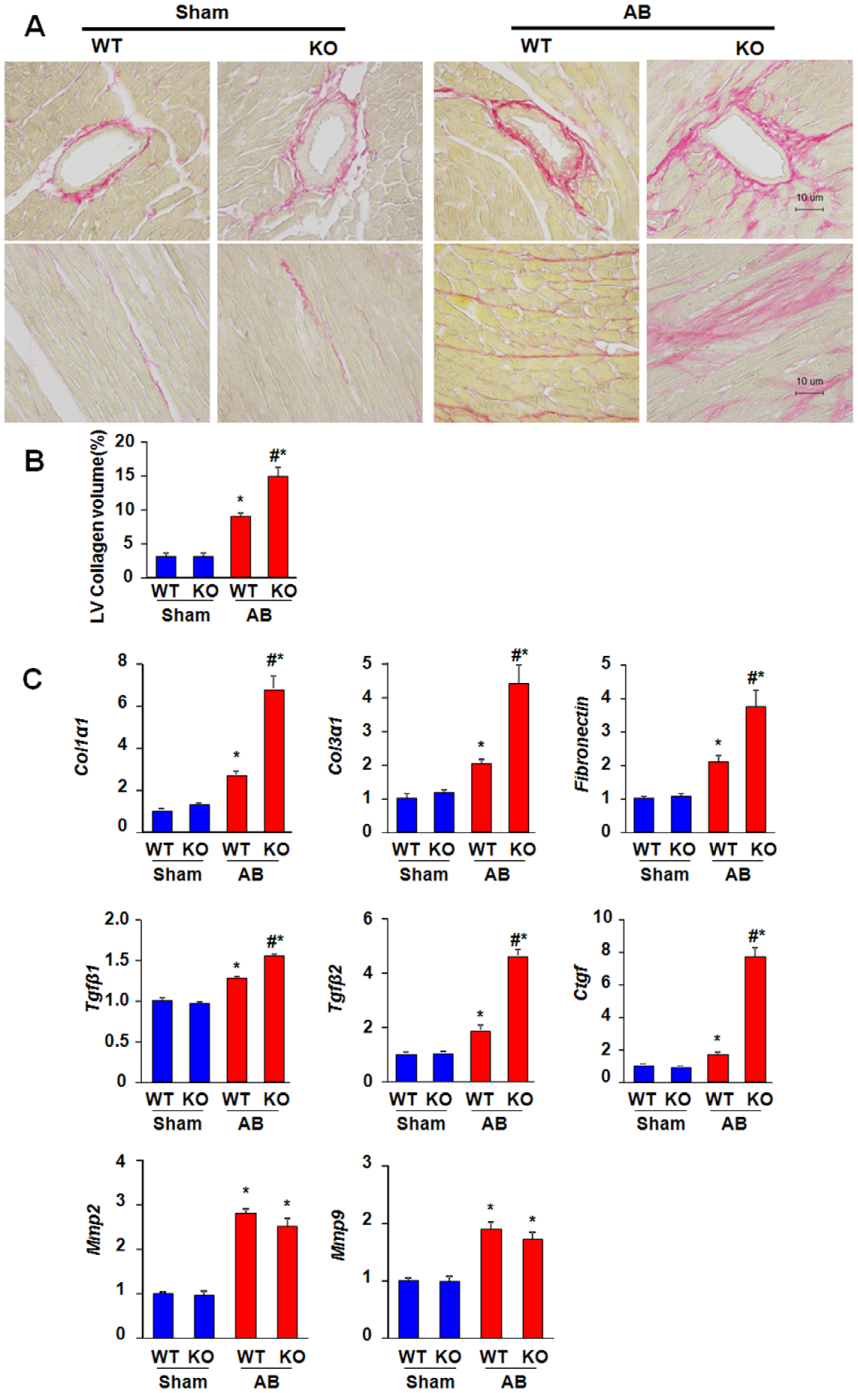
Increased fibrosis in ELTD1 −/− mice. A and B, PSR staining and quantitative fibrotic area measurements. Data represent as mean±SEM (n = 8). C RT-PCR analyses of *Tgfβ1, Tgfβ2, Colα1, Co3α1, Ctgf, Mmp2, and Mmp9* mRNA levels 28 days after surgeries. GAPDH was used as the sample loading control. (n = 6) *P<0.01 vs. corresponding sham; #P<0.01 vs. WT/AB after AB.

Fibrosis is an important aspect of cardiac hypertrophy that is characterized by extracellular matrix (ECM) deposition and is mediated by various cytokines [8]. In our study, PSR staining revealed striking perivascular and interstitial fibrosis in ventricles in response to AB, and the extent of cardiac fibrosis remarkably increased in the KO mice (Figure 3A, B).

We tried to elucidate the underlying mechanism for exacerbated fibrosis. The mRNA levels of pro-collagen type I α1 (*Col1α1*), pro-collagen type III α1 (*Col3α1*), and fibronectin were significantly higher in KO mice than in WT mice after AB (Figure 3C), revealing an elevated deposition of connective tissue in the ventricles. Furthermore, mediators of fibrosis (including transforming growth factor-β1 (*Tgfβ1*), *Tgfβ2*, and connective tissue growth factor (*Ctgf*) demonstrated exaggerated responses in KO mice relative to WT after AB, as indicated by mRNA level analysis (Figure 3C). In contrast, the level of matrix metalloproteinase 2(*Mmp2*) and *Mmp9* increased similarly after AB in both groups (Figure 3C).

### Effect of ELTD1 on MAPKs and Akt Activation

**Figure 4:**
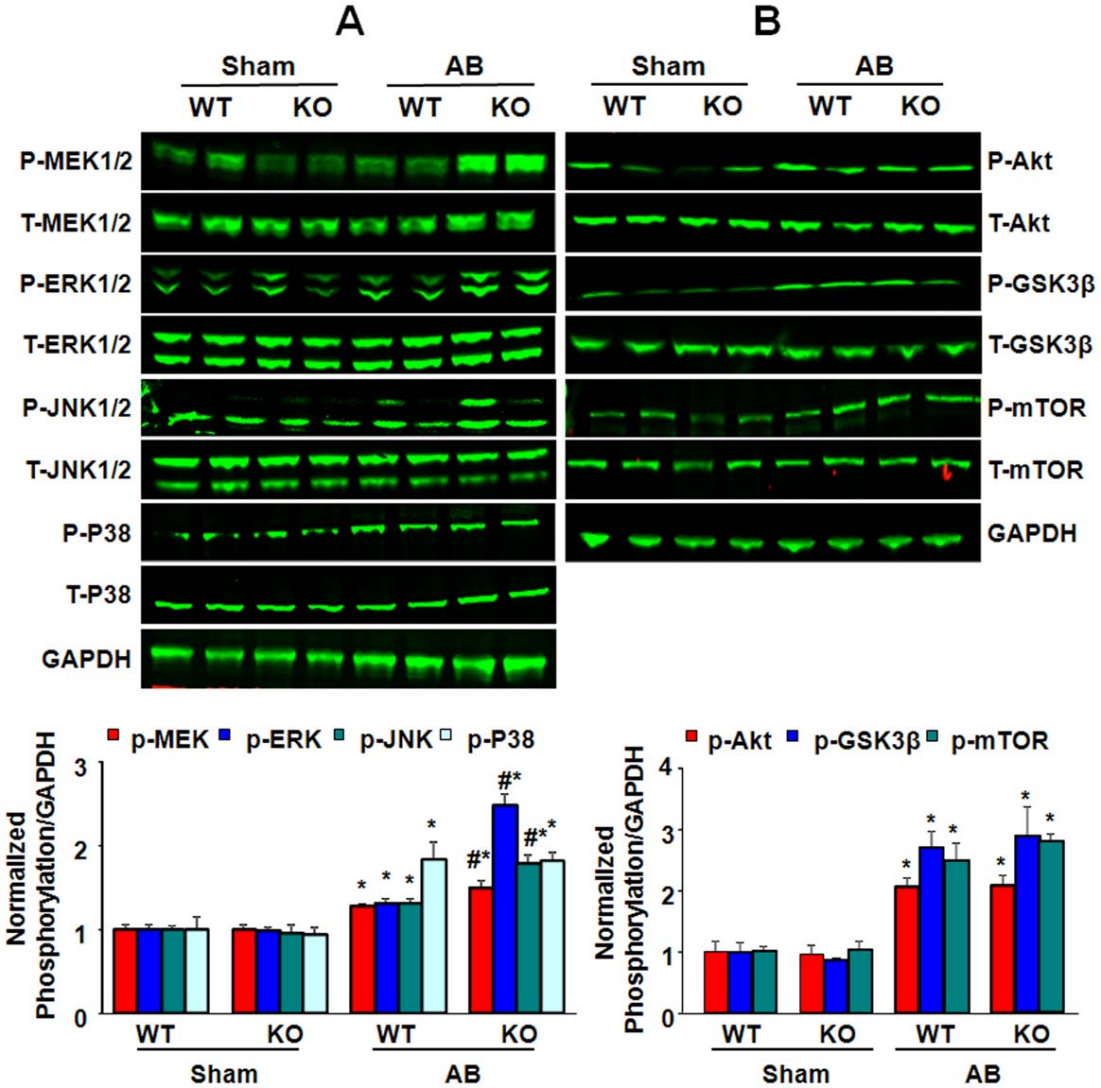
Effect of ELTD1 on MAPKs and PI3K –Akt signaling pathway. A, Western blots of MEK1/2, ERK1/2, p38, and JNK phosphorylation and their total protein levels 28 days after surgery in WT and KO mice. GAPDH was used as the sample loading control. (n = 6). Up, representative blots. Down, quantitative results. B, Western blots of Akt,GSK3β, and mTOR phosphorylation and their total protein levels 28 days after surgery. GAPDH was used as the sample loading control (n = 6). Up, representative blots. Down, quantitative results. For A and B, Data represent as the means ± SEM *P<0.01 vs. corresponding sham; #P<0.01 vs. WT/AB after AB.

MAPKs and PI3K-Akt are among the most thoroughly characterized signaling pathways that are activated by pressure overload and drive cardiac hypertrophy [9], [10]. Therefore, we focused our analysis following AB on defining changes in signaling events that precede unfavorable remodeling. As shown in Figure 4A, AB promoted the phosphorylation of MEK1/2-ERK1/2, JNK1/2, and p38-MAPK in KO and WT mice. Deletion of ELTD1 further increased activation of MRK1/2-ERK1/2 and JNK1/2 after AB. Notably, the phosphorylation of p38-MAPK was not affected by the absence of ELTD1. Our data showed that the activation of Akt was amplified by AB, but no difference was found between the two groups after AB (Figure 4B). In addition, we examined the activation of the downstream effector of Akt but found no difference in GSK3β and mTOR phosphorylation between the two groups after AB (Figure 4B).

## Discussion

Cardiac hypertrophy and subsequent heart failure are important clinical determinants of morbidity and mortality [11]. In the present study, we demonstrated for the first time that ELTD1 deficiency exacerbated cardiac hypertrophy and cardiac function impairment in vivo using genetically engineered ELTD1 KO mice in AB-induced pressure overload model.

In accordance with ELTD1’s expression in rats and humans [4], ELTD1 is highly expressed in mouse hearts. Dilated evolution of hypertrophic cardiomyopathy is characterized by wall thinning and cavity dilatation. This dilated status with end stage heart failure is one single most frequent indication for heart transplantation [12], and represents decompensated remodeling of the heart to external stimuli and mechanical stresses [13].The expression of ELTD1 drastically changed in failing human and murine ventricles after AB, suggesting involvement of ELTD1 in pathological hypertrophy.

ELTD1 deficiency exacerbates maladaptive remodeling induced by pressure overload. Three major sets of experimental observations support this conclusion. First, an in vivo study demonstrated that ablation of the ELTD1 gene leads to a striking phenotype: an approximately 27% increase in HW/BW, a 29% increase in CSA, and a 65% increase in fibrosis area relative to age- and body weight-matched WT animals after AB. The hypertrophic markers are the second line of independent evidence supporting ELTD1’s involvement in hypertrophy. It’s commonly accepted that there are increased expressions of *Anp*, *Bnp*, *β-MHC*, *Acta1*, and decreased expressions of *Serca2α* and *α-MHC* in mice subjected to AB compared to controls [1]. In this study, KO of ELTD1 further increased the expressions of *Anp*, *Bnp*, *β-MHC*, *Acta1* and decreased the expressions of *Serca2α* and *α-MHC* in mice subjected to AB. Third, KO of ELTD1 led to severely impaired cardiac function accompanied by greater LV dilation, as evidenced by echocardiography and PV loop measurements. Myocardial fibrosis and myocytes hypertrophy have been implicated in increased myocardial stiffness, resulting in diastolic dysfunction. SERCA activity not only influences the rate of cardiac relaxation but also the amount of Ca^2+^ available to activate the next contraction [14], which might be an underlying mechanism of the loss of function.

Cardiac fibrosis, which is result from unbalanced synthesis and degradation of collagens or/and other ECM components, is an important hallmark of maladaptive hypertrophy [8]. Morphology staining of PSR exhibited enlarged collagens in the myocardium of KO-AB mice and was further confirmed by PCR analysis of *Col1α1*, *Col3α1*, and *Fibronectin*. *Tgfβ* and its pro-fibrotic target gene *Ctgf* can promote ECM production [15], [16], [17]. Increased synthesis of ECM components increases the levels and activities of enzymes (MMPs), and MMPs degrade the ECM to counterbalance the previous ECM production [18]. This is a benignant circle. In our study, ELTD1 deficiency magnified the function of *Tgfβ1* and *Ctgf*, which promotes the deposition of collagen. PCR analyses of *Mmp2, 9* revealed similarly elevated mRNA levels of them in KO and WT mice after AB. These results mean that KO of ELTD1 promoted the synthesis of collagen but did not augment the MMP -related degradation accordingly. Thus, the mechanism of the pro-fibrotic action of ELTD1 can be attributed both to the inhibition of degradation and promotion of synthesis.

MAPKs and PI3K-Akt signaling were investigated in our study to determine the possible molecular mechanisms of the pro-hypertrophy action of ELTD1. All three types of MAPKs are activated in response to GPCR agonists and mechanical stress; in addition, these MAPKs are activated in pressure-overloaded hearts and failing human hearts [19], [20], [21]. Activation of ERK1/2 by MEK1/2 phosphorylation leads to two fundamental changes [22], [23]. First, the subsequent phosphorylation of substrates may contribute to cell hypertrophy by increasing protein synthesis or hypertrophy-related gene expression [23], [24]. Second, auto-phosphorylation of ERK1/2 results in nuclear translocation, allowing ERK1/2 to phosphorylate nuclear targets, which in turn promotes the transcription of hypertrophic genes, as shown by biopsies and a transgenic mouse model [23]. Both these processes may contribute to the maladaptive phenotype associated with pathological hypertrophic responses. Various groups have suggested that JNK is an important regulator of pathological hypertrophy. However, different studies have reported that the inactivation of JNK induced by the loss of functional MEK4 (upstream of JNK) led to attenuated or enhanced hypertrophy in pressure-overloaded models [25], [26]. JNK is also considered to be involved in the increased expression of ANP, TGFβ, Col1α1, and Col3α1during cardiac hypertrophy [27]. According to our study, JNK, ERK1/2, and MEK1/2, the upstream activator of ERK1/2, were activated after pressure overload, and the KO of ELTD enhanced the increased phosphorylation of MEK-ERK1/2 and JNK after AB. Activation of p38 led to increased expression of the fetal gene program, substantial induction of interstitial fibrosis, and the loss of contractility [28]. The results of many p38 studies appear to be contradictory: the lack of expression of expression of p38α or its upstream regulators MEK3 and MEK6 in a transgenic model led to a significant increase in heart size; whereas, negative expression of p38β led to a lack of cardiac hypertrophy but reduced systolic function [29], [30]. These results suggest an important role of p38 in pressure overload-induced remodeling. We found increased phosphorylation of p38 in the AB group relative to the sham-operated group, in agreement with Sari et al. [31] and Cai et al. [32]. We did not explore the activation of different isoforms, and the phosphorylation of different p38 isoforms seems to be influenced by race and time [33]. No difference in p38 phosphorylation was found in ELTD1 KO mice relative to WT mice in our research. The PI3K (p110α)-Akt pathway has been demonstrated by a genetic mouse model to play a critical role in regulating cardiac hypertrophy. Although PI3K-Akt is thought to regulate physiological hypertrophy [18], sustained or marked activation of Akt alters the expression profile to that typical of a pathological state [34]. We found activated phosphorylation of Akt, GSK3β and mTOR in the AB group relative to the sham-operated group, but no difference between the KO and WT mice. Our results suggest that ELTD1 promotes cardiac hypertrophy through MEK-ERK1/2 and JNK signaling rather than through PI3K-Akt signaling. However, further experiments are needed to determine whether ELTD1 interferes with MAPKs and Akt signaling in the same way in response to stimulus other than pressure overload, and to understand the exact molecular connection by which ELTD1 regulates the ERK and JNK pathways.

In conclusion, the findings of the present study show a key role for ELTD1 in regulating the pathological hypertrophy of the heart, likely through its activating effects on the downstream MER1/2-ERK1/2 and JNK signaling cascades. These results provide new insight into the pathogenesis of cardiac remodeling. However, additional studies are needed to test whether modification of ELTD1 function might improve the clinical outcome in cases of human cardiac hypertrophy or other cardiac diseases.

## References

[1] Bernardo BC, Weeks KL, Pretorius L, McMullen JR (2010). Molecular distinction between physiological and pathological cardiac hypertrophy: Experimental findings and therapeutic strategies.. Pharmacol Ther.

[2] Belmonte SL, Blaxall BC (2011). G protein coupled receptor kinases as therapeutic targets in cardiovascular disease.. Circ Res.

[3] Zhang P, Mende U (2011). Regulators of G-protein signaling in the heart and their potential as therapeutic targets.. Circ Res.

[4] Nechiporuk T, Urness LD, Keating MT (2001). ETL, a novel seven-transmembrane receptor that is developmentally regulated in the heart. ETL is a member of the secretin family and belongs to the epidermal growth factor-seven-transmembrane subfamily.. J Biol Chem.

[5] Luttrell LM, van Biesen T, Hawes BE, Koch WJ, Krueger KM (1997). G-protein-coupled receptors and their regulation: activation of the MAP kinase signaling pathway by G-protein-coupled receptors.. Adv Second Messenger Phospho protein Res.

[6] Pak Y, O’Dowd BF, Wang JB, George SR (1999). Agonist-induced, G Protein-dependent and -independent down-regulation of the μ Opioid Receptor.. J Biol Chem.

[7] Li H, He C, Feng J, Zhang Y, Tang Q (2010). Regulator of G protein signaling 5 protects against cardiac hypertrophy and fibrosis during biomechanical stress of pressure overload.. Proc Natl Acad Sci U S A.

[8] Creemers EE, Pinto YM (2011). Molecular mechanisms that control interstitial fibrosis in the pressure-overloaded heart.. Cardiovasc Res.

[9] Proud CG (2004). Ras, PI3-kinase and mTOR signaling in cardiac hypertrophy.. Cardiovasc Res.

[10] Rose BA, Force T, Wang Y (2010). Mitogen-activated protein kinase signaling in the heart: angels versus demons in a heart-breaking tale.. Physiol Rev.

[11] Kubo T, Gimeno JR, Bahl A, Steffensen U, Steffensen M (2007). Prevalence, clinical significance, and genetic basis of hypertrophic cardiomyopathy with restrictive phenotype.. J Am Coll.

[12] Spirito P, Seidman CE, McKenna WJ, Maron BJ (1997). The management of hypertrophic cardiomyopathy.. N Engl J Med.

[13] Biagini E, Coccolo F, Ferlito M, Perugini E, Rocchi G (2005). Dilated-hypokinetic evolution of hypertrophic cardiomyopathy: prevalence, incidence, risk factors, and prognostic implications in pediatric and adult patients.. J Am Coll Cardiol.

[14] Inesi G, Prasad AM, Pilankatta R (2008). The Ca2+ ATPase of cardiac sarcoplasmic reticulum: Physiological role and relevance to diseases.. Biochem Biophys Res Commun.

[15] Brooks WW, Conrad CH (2000). Myocardial fibrosis in transforming growth factor β1 heterozygous mice.J Mol Cell Cardiol.

[16] Rosenkranz S, Flesch M, Amann K, Haeuseler C, Kilter H (2002). Alterations of β-adrenergic signaling and cardiac hypertrophy in transgenic mice overexpressing TGF-β1.. Am J Physiol Heart Circ Physiol.

[17] Panek AN, Posch MG, Alenina N, Ghadge SK, Erdmann B (2009). Connective tissue growth factor overexpression in cardiomyocytes promotes cardiac hypertrophy and protection against pressure overload.. PLoS One.

[18] Berk BC, Fujiwara K, Lehoux S (2007). ECM remodeling in hypertensive heart disease.. J Clin Invest.

[19] Purcell NH, Wilkins BJ, York A, Saba-El-Leil MK, Meloche S (2007). Genetic inhibition of cardiac ERK1/2 promotes stress-induced apoptosis and heart failure but has no effect on hypertrophy in vivo.. Proc Natl Acad Sci U S A.

[20] Rose BA, Force T, Wang Y (2010). Mitogen-activated protein kinase signaling in the heart: angels versus demons in a heart-breaking tale.. Physiol Rev.

[21] Xiao JF, Moon M, Yan L, Nian M, Zhang Y (2012). Cellular FLICE-inhibitory protein protects against cardiac remodelling after myocardial infarction.. Basic Res Cardiol.

[22] Robbins DJ, Zhen E, Owaki H, Vanderbilt CA, Ebert D (1993). Regulation and properties of extracellular signal-regulated protein kinases 1 and 2 in vitro.. J Biol Chem.

[23] Lorenz K, Schmitt JP, Schmitteckert EM, Lohse MJ (2009). A new type of ERK1/2 autophosphorylation causes cardiac hypertrophy.. Nat Med.

[24] Karin M (1996). The regulation of AP-1 activity by mitogen-activated protein kinases.. Philos Trans R Soc Lond B Biol Sci.

[25] Liu W, Zi M, Jin J, Prehar S, Oceandy D (2009). Cardiac-specific deletion of MKK4 reveals its role in pathological hypertrophic remodeling but not in physiological cardiac growth.. Circ.

[26] Choukroun G, Hajjar R, Fry S, Monte Fd, Haq S (1999). Regulation of cardiac hypertrophy in vivo by the stress-activated protein kinases/c-Jun NH2-terminal kinases.. J Clin Invest.

[27] Kim S, Iwao H (1999). Activation of mitogen-activated protein kinases in cardiovascular hypertrophy and remodeling.. Jpn J Pharmacol.

[28] Liao P, Georgakopoulos D, Kovacs A, Zheng M, Lerner D (2001). The in vivo role of p38 MAP kinases in cardiac remodeling and restrictive cardiomyopathy.Proc Natl Acad Sci U S A.

[29] Zhang S, Weinheimer C, Courtois M, Kovacs A, Zhang C (2003). The role of the Grb2–p38 MAPK signaling pathway in cardiac hypertrophy and fibrosis.. J Clin Invest.

[30] Braz JC, Bueno OF, Liang Q, Wilkins BJ, Dai YS (2003). Targeted inhibition of p38 MAPK promotes hypertrophic cardiomyopathy through upregulation of calcineurin-NFAT signaling.. J Clin Invest.

[31] Sari FR, Widyantoro B, Thandavarayan RA, Harima M, Lakshmanan AP (2011). Attenuation of CHOP-mediated myocardial apoptosis in pressure-overloaded dominant negative p38α mitogen-activated protein kinase mice.. Cell Physiol Biochem.

[32] Cai W-F, Zhang X-W, Yan H-M, Ma Y-G, Wang X-X (2010). Intracellular or extracellular heat shock protein 70 differentially regulates cardiac remodelling in pressure overload mice.. Cardiovasc Res.

[33] Dingar D, Merlen C, Grandy S, Gillis M-A, Villeneuve LR (2010). Effect of pressure overload-induced hypertrophy on the expression and localization of p38 MAP kinase isoforms in the mouse heart.. Cell Signal.

[34] Matsui T, Li L, Wu J, Cook S, Nagoshi T (2002). Phenotypic spectrum caused by transgenic overexpression of activated Akt in the heart.. J Biol Chem.

